# CD146 is required for VEGF-C-induced lymphatic sprouting during lymphangiogenesis

**DOI:** 10.1038/s41598-017-06637-7

**Published:** 2017-08-07

**Authors:** Huiwen Yan, Chunxia Zhang, Zhaoqing Wang, Tao Tu, Hongxia Duan, Yongting Luo, Jing Feng, Feng Liu, Xiyun Yan

**Affiliations:** 1 Key Laboratory of Protein and Peptide Pharmaceuticals, Institute of Biophysics, Chinese Academy of Sciences, Beijing, 100101 China; 2 State Key Laboratory of Membrane Biology, Institute of Zoology, Chinese Academy of Sciences, Beijing, 100101 China; 3 College of Life Sciences, University of Chinese Academy of Sciences, Beijing, 100049 China

## Abstract

VEGF-C is essential for lymphangiogenesis during development and tumor progression. VEGFR-3 is the well-known cognate receptor of VEGF-C to regulate lymphatic migration and proliferation, but the receptor of VEGF-C in regulating lymphatic sprouting, the initiating step of lymphangiogenesis, still remains elusive. Here we use both *in vitro* and *in vivo* methods to demonstrate CD146 as a receptor of VEGF-C to regulate lymphangiogenesis, especially at the sprouting step. Mechanistically, CD146 selectively activates the downstream p38 kinase, upon VEGF-C stimulation, to regulate lymphatic sprouting. Moreover, CD146 can also activate ERK to mediate VEGF-C regulation of the subsequent proliferation and migration of lymphatic endothelial cells. In zebrafish embryos, knockdown or dysfunction of CD146 results in similar developmental defects in lymphatic sprouting, capillary network, parachordal lymphangioblast (PL), and thoracic duct (TD) similar to down-regulation of VEGF-C. Altogether, our data reveals a critical role of CD146 to mediate VEGF-C signaling pathway in lymphangiogenesis.

## Introduction

The lymphatic vascular network plays indispensable physiological roles in homeostasis, metabolism and immunity^1^. Lymphangiogenesis refers to the formation of lymphatic vessels from the pre-existing veins^2, 3^, and involves signaling pathways that coordinately regulate sprouting, morphogenesis, and the ultimate formation of lymphatic vessel network from lymphatic endothelial cells (LECs)^4^. Sprouting or budding of LECs from pre-existing veins is the initial step of lymphangiogenesis^5^. VEGF-C is one of the major pro-lymphangiogenesis inducers and controls the whole process of lymphangiogenesis, including the initiation of lymphatic sprouting^6–8^. Despite extensive studies on VEGF-C functions in lymphangiogenesis^8, 9^, the central question of which receptors mediate VEGF-C signal transduction for lymphatic sprouting remains unaddressed.

It has been reported that VEGF-C regulates proliferation and migration of LECs by VEGFR3 via activation of the ERK/MAPK and PI3K/AKT pathways^10–12^. Ectopic *vegfc* expression causes lymphatic hyperplasia^13^, whereas *vegfc* deficiency causes destructive sprouts of LECs and loss of primary lymph sacs^8^. *vegfr3* deficient mice die at midgestation (E10.5) due to the failure of the remodeling of primary vascular network^14^. To investigate the role of VEGFR3 in lymphatic vascular development, a conditional knockout model targeting the ligand binding domain of VEGFR3 was generated. Surprisingly, LECs are able to sprout from the cardinal veins and form round-shaped lymph sacs^15^. Thus, the distinct phenotypes of VEGF-C and VEGFR-3 knockout mice indicate a possibility of the existence of other VEGF-C receptors that account for lymphatic sprouting.

CD146 is a cell adhesion molecule which contains five Ig-like domains in its extracellular region, an ERM (protein complex of ezrin, radixin and moesin) binding site in its cytoplasmic tail and a single transmembrane domain. CD146 is widely known as a biomarker of angiogenesis^16^ and participates in VEGF-A signaling, a major inducer of pro-angiogenesis, to facilitate arterial development^17^. Intriguingly, many factors involved in arterial development also control lymphatic development, such as the forkhead transcription factors Foxc1/2^18, 19^, EphrinB2^20^ and Dll4/Notch signaling^21^. Therefore, it is likely that CD146 may also function in lymphangiogenesis.

Here we report for the first time that CD146, a biomarker of angiogenesis, acts as a novel receptor of VEGF-C to control lymphatic sprouting in lymphangiogenesis. In this study, we investigated the role of CD146 in lymphangiogenesis as well as lymphatic system development by using *in vitro* and *in vivo* models. Our results suggest that VEGF-C/CD146 pathway is a critical lymphatic development signaling to coordinate the concerted activation of p38 and ERK for decoding VEGF-C signals into fine-tuned signaling output in lymphangiogenesis, especially the key step of lymphatic sprouting.

## Results

### CD146 controls LEC sprouting *in vitro*

**Figure 1:**
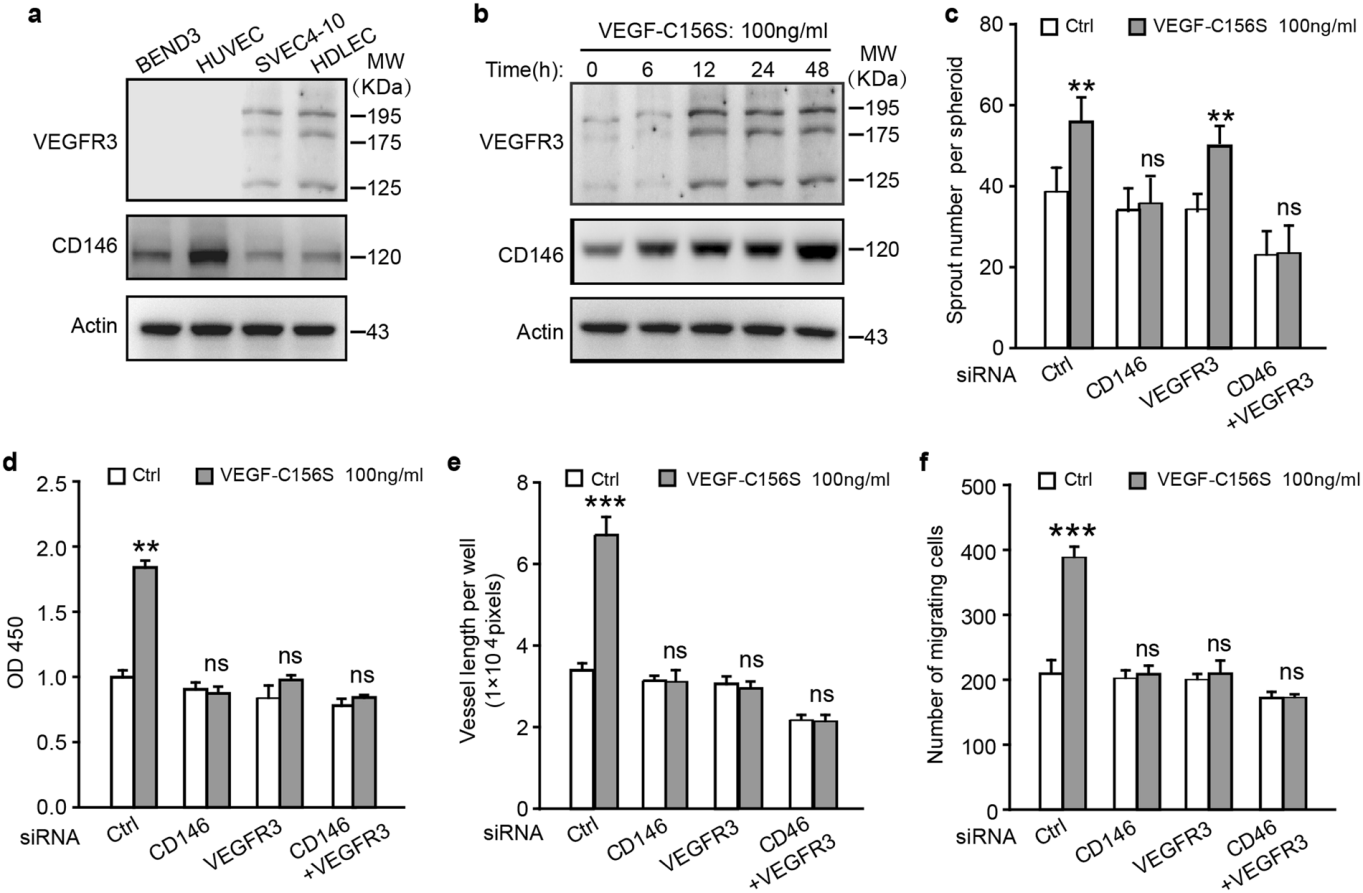
CD146 is expressed in LEC and regulates LEC activation induced by VEGF-C. (**a**) WB analysis of CD146 and VEGFR-3 expression in four different endothelial cell lines. (**b**) Serum-starved HDLEC cells were treated with VEGF-C156S (100 ng/ml) for different time intervals. The expression of CD146 and VEGFR-3 was determined by WB. (**c**–**f**) HDLEC cells, transfected with siRNA of control, CD146, VEGFR-3 or combination of CD146 and VEGFR-3, were subjected to spheroid sprouting assay (**c**), proliferation assay (d), tube formation assay (**e**) and transwell migration assay (**f**). VEGF-C156S was applied at the indicated concentration. Data were expressed as means ± SEM from 3 independent experiments (n = 3 in each group of **c**–**f**). Significant difference was determined by Student’s *t-*test (*P < 0.05; **P < 0.01; ***P < 0.001; ns, no significant difference). Full-length blots are presented in Supplementary Fig. S4.

To test whether CD146 is involved in lymphangiogenesis, we firstly sought to examine whether CD146 is endogenously expressed in lymphatic cells. Primary human dermal lymphatic endothelial cells (HDLECs) and mouse endothelial cell line SVEC4-10 are LEC lines that express LEC specific markers^22^, and our western blotting (WB) results showed that both CD146 and VEGFR-3 are expressed in these two LEC lines. As control, two blood endothelial cell lines including human umbilical vein endothelial cells (HUVECs) and mouse brain endothelial cell line BEND3 only express CD146 but not VEGFR-3 (Fig. 1a), which is consistent with previous report^23^. Since HDLEC line expresses endogenous CD146, it was selected for further *in vitro* functional investigations. We also found that similar to VEGFR-3, CD146 expression was enhanced by VEGF-C156S, a mutant form of VEGF-C, which binds VEGFR-3 but not VEGFR-2 (Figs 1b and S1)^24^. These results imply that CD146 may involve in VEGF-C-induced lymphangiogenesis.

To investigate the role of CD146 in VEGF-C156S-induced LEC activation, we knocked down CD146 using RNA interference (RNAi) technology. Interestingly, we found that down-regulation of CD146 significantly reduced VEGF-C induced LEC activities including sprouting, proliferation, migration and tube formation. In contrast, VEGFR-3 deficiency inhibited proliferation, migration and tube formation but not sprouting of LEC (Figs 1c–f and S1c). These *in vitro* results demonstrated that CD146, but not VEGFR-3, is involved in the sprouting process, while both CD146 and VEGFR-3 are crucial for other functions of LECs, suggesting that CD146 plays a distinct role in lymphangiogenesis.

### CD146 mediates p38 pathway induced by VEGF-C

**Figure 2:**
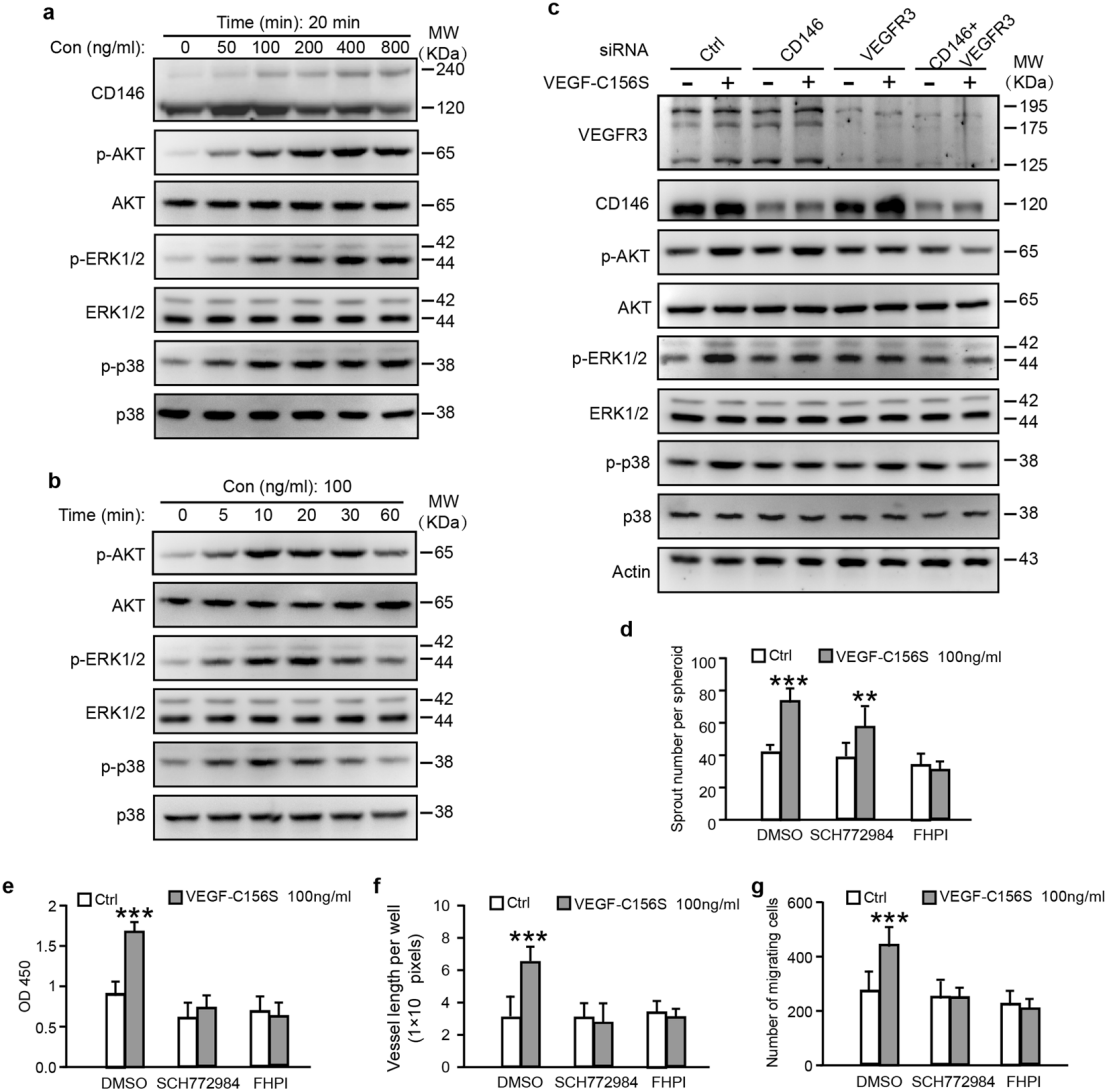
VEGF-C elicitation of different signaling cascades downstream of CD146 or VEGFR-3. (**a**,**b**) Dose and time effect of VEGF-C156S on HDLEC cells. Serum-starved cells were treated with VEGF-C156S for 20 min at different concentrations in (**a**) or with 100 ng/ml of VEGF-C156S at different time intervals in (**b**). Dimerization of CD146 and phosphorylation and expression of AKT, ERK and p38, elicited from VEGF-C156S, were analyzed by WB. (**c**) HDLEC cells, transfected with siRNA of control, CD146, VEGFR-3 or combination of CD146 and VEGFR-3, were treated with 100 ng/ml of VEGF-C156S for 20 min. (**d**–**g**) HDLEC cells, pretreated with SCH772984 or FHPI for 72 hr, were subjected to spheroid sprouting assay (**d**), proliferation assay (**e**), tube formation assay (**f**) and transwell migration assay (**g**). VEGF-C156S was applied at the indicated concentration. Data were expressed as means ± SEM from 3 independent experiments (n = 12 in each group of **d**–**g**). Significant difference was determined by Student^’^s *t-*test (*P < 0.05; **P < 0.01; ***P < 0.001; ns, no significant difference). Full-length blots are presented in Supplementary Fig. S4.

To delineate downstream pathways mediating VEGF-C-induced lymphatic endothelial cell activation, we first examined the dimerization of CD146, an important mechanism required for its downstream signaling. In HDLECs, VEGF-C induced CD146 dimerization in a dose-dependent manner (Fig. 2a). AKT and ERK are two major downstream signaling cascades of VEGF-C in LECs^10^. We found that in addition to AKT and ERK, p38 was also activated by VEGF-C156S in a dose- and time-dependent manner (Figs 2a,b and S2a,b). Furthermore, siRNA knockdown of CD146 significantly reduced phosphorylation levels of p38 and ERK but not of AKT. In contrast, knockdown of VEGFR-3 significantly reduced phosphorylation levels of AKT and ERK without affecting p38 (Fig. 2c). The quantified phosphorylation levels of AKT, ERK and p38 were shown in Fig. S2C–E. To further define the specific downstream signaling of CD146 and VEGFR-3. HEK293 cells, which do not express endogenous CD146 and VEGFR-3, were selected and exogenously transfected with CD146 or VEGFR-3 expressing plasmids. Consistent with the results of knockdown of CD146 or VEGFR-3 in LECs, over-expression of VEGFR-3 significantly augmented VEGF-C156S-induced phosphorylation levels of AKT and ERK but not of p38; while over-expression of CD146 significantly enhanced phosphorylation levels of p38 and ERK but not of AKT (Fig. S3). To further confirm that CD146 regulates LECs ability via MAPK/ERK and p38 signaling, we used ERK1/2 inhibitor, SCH772984 and p38 inhibitor, FHPI to test the effects of these drugs on LECs sprouting, proliferation, migration and tube formation. It showed that both inhibitors reduced proliferation, migration and tube formation efficiently but only FHPI blocked sprouting induced by VEGF-C (Fig. 2d–g).

Thus, these results demonstrated that in response to VEGF-C156S stimulation, p38 and ERK are the dominant signaling cascades downstream of CD146. Furthermore, CD146 regulates sprouting via p38 pathway.

### CD146 interacts with VEGF-C directly

**Figure 3:**
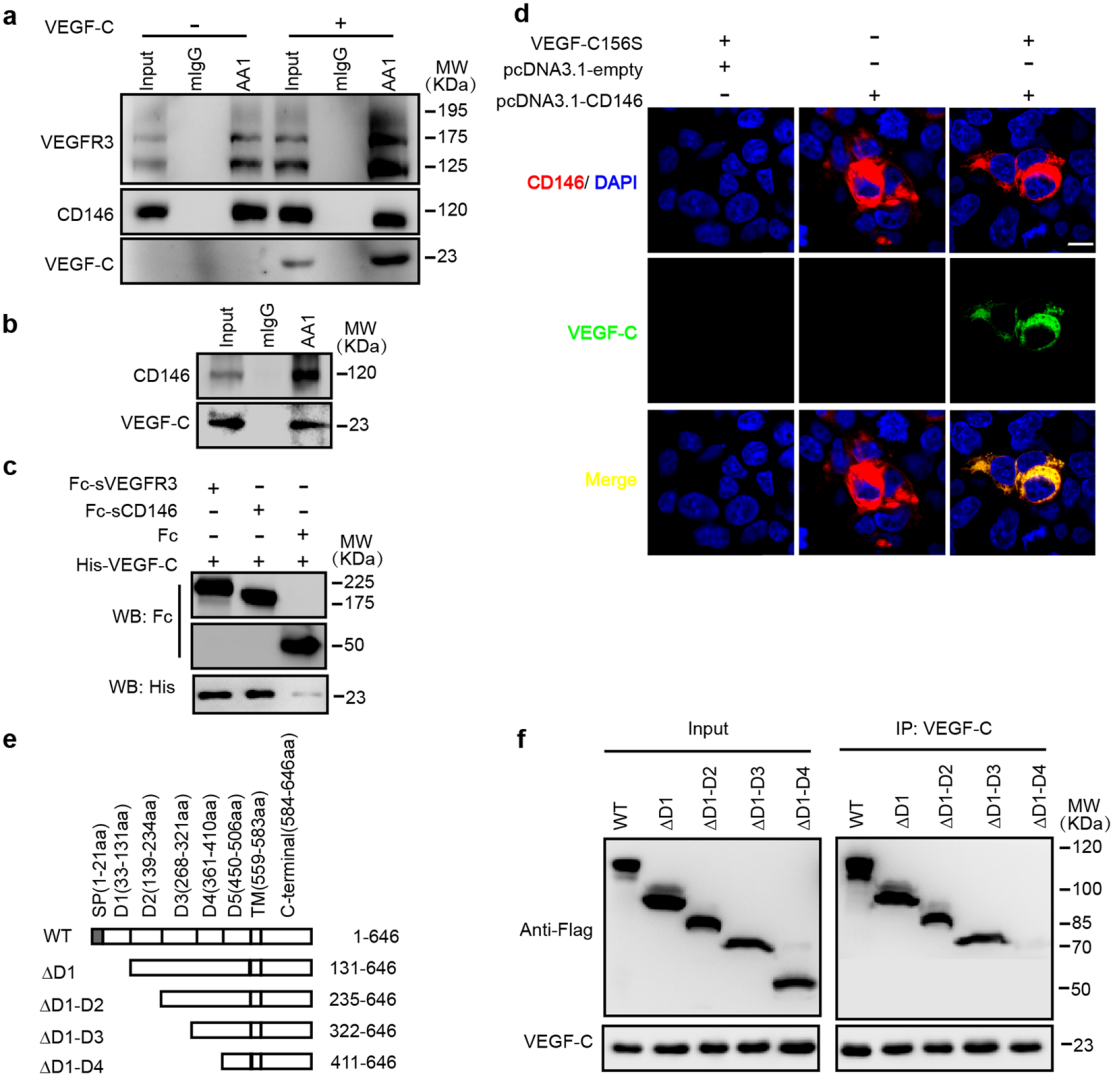
CD146 directly binds to VEGF-C. (**a**) Co-immunoprecipitation assays in HDLECs. Cells were incubated with normal culture medium or VEGF-C156S (100 ng/ml) conditional medium. The cell lysates were prepared for immunoprecipitation with control mIgG or anti-CD146 mAb AA1. (**b**,**d**) HEK293 cells transfected with control empty or CD146 expression vectors were incubated with VEGF-C conditional medium or transfected with CD146 encoding vector without VEGF-C incubation. Binding of VEGF-C-His to cells was detected by co-immunoprecipitation (**b**) and immunofluorescence (**d**). Scale bars: 100 μm. (**c**) Direct *in vitro* interaction between VEGF-C and CD146 proteins. Purified Fc, Fc-CD146 or Fc-VEGFR-3 (200 ng/ml) was incubated with VEGF-C protein (200 ng/ml). The results were analyzed by WB. (**e**,**f**) defining the domain of CD146 required for interaction with VEGF-C. CD146 and its truncation mutants were expressed as Flag-tagged proteins. Immunoprecipitation was performed using anti-VEGF-C mAb. Full-length blots are presented in Supplementary Fig. S4.

To test the relationship between CD146 and VEGF-C, we first performed co-immunoprecipitation (co-IP) assay in LECs to test the potential interaction between CD146 and VEGF-C. Both VEGFR-3 and VEGF-C were immunoprecipitated by anti-CD146 antibody but not by mouse IgG (mIgG). Pre-incubation of the cells with VEGF-C enhanced the amount of immunoprecipitated VEGFR-3 and VEGF-C (Fig. 3a). The interaction between CD146 and VEGF-C was further confirmed by co-IP assay in HEK293 cells transfected with plasmids encoding CD146 and incubation with conditional media of VEGF-C (Fig. 3b). To test whether the interaction between VEGF-C and CD146 is direct or indirect, we used purified proteins of the Fc-fragment of human IgG (negative control), the fusion proteins of Fc-CD146 and Fc-VEGFR-3 to conduct a pull-down assay. VEGF-C bound with either Fc-CD146 or Fc-VEGFR-3 but not with Fc, indicating that similar to VEGFR-3, CD146 is capable of binding with VEGF-C directly (Fig. 3c). In addition, to test whether the VEGF-C-CD146 interaction could occur on the surface of living cells, we performed cell surface binding assay. After transfection of HEK293 cells with plasmids encoding CD146, cells were incubated with conditional media of VEGF-C. Immunofluorescence was performed to detect the signals of CD146 and VEGF-C. It showed that positive signals were detected on the cells expressing CD146 (Fig. 3d), supporting a direct interaction between CD146 and VEGF-C on the cell surface.

We next mapped the domains required for VEGF-C-CD146 interaction using a series of CD146 truncation mutant constructs with the extracellular Ig domains deleted sequentially. In the co-immunoprecipitation experiments, VEGF-C was immunoprecipitated by the full-length CD146, or the truncation mutants, ∆D1, ∆D1-D2 and ∆D1-D3. However, a further deletion to remove the 4^th^ Ig domain (∆D1-D4) abolished the interaction (Fig. 3e and f), indicating the 4^th^ Ig domain of CD146 is critical for the binding to VEGF-C.

Together, these results demonstrated a direct interaction between CD146 and VEGF-C, indicating that CD146 is a novel receptor of VEGF-C.

### CD146 cytoplasmic domain is required for LEC activation induced by VEGF-C

**Figure 4:**
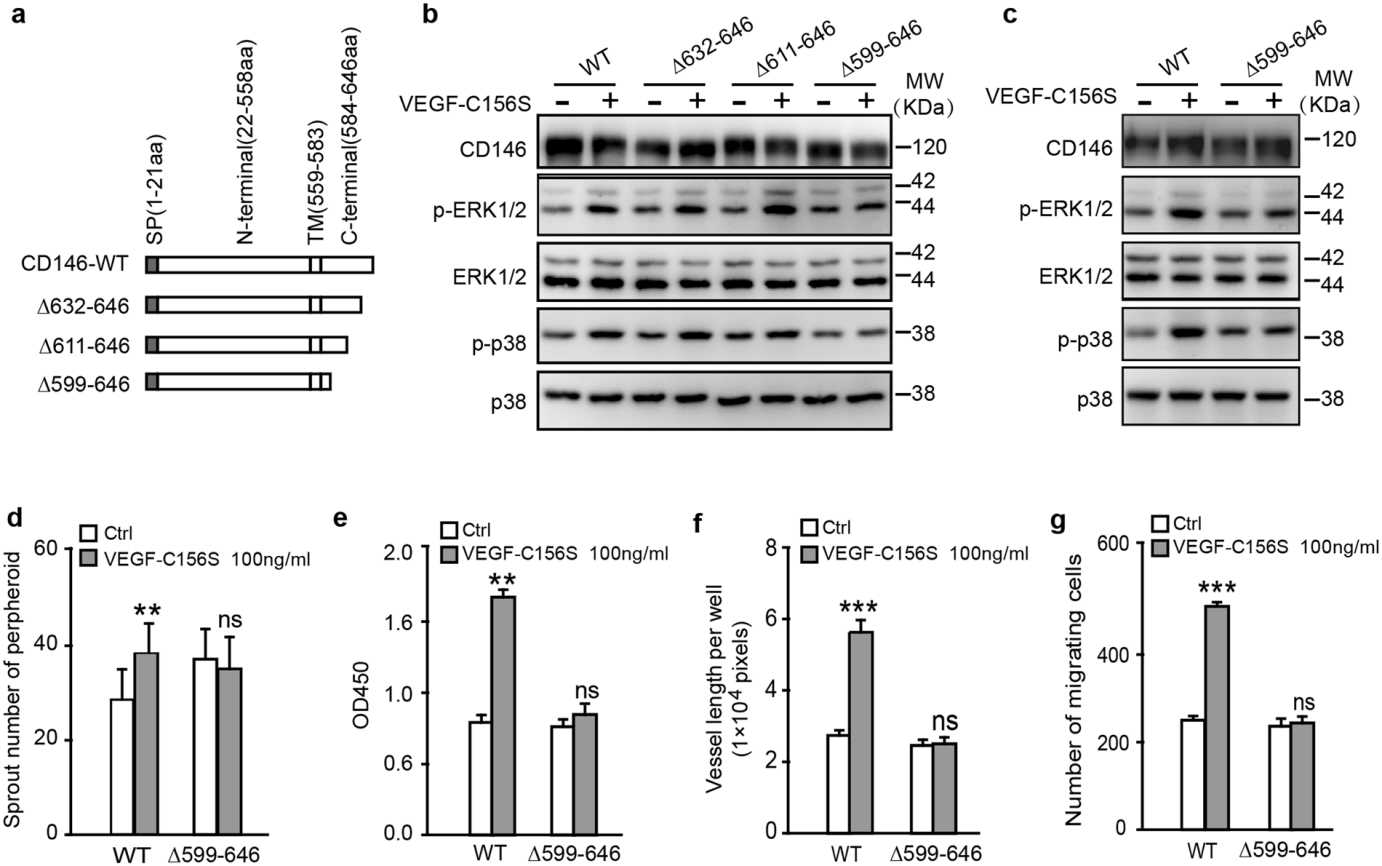
Functional cytoplasmic domain of CD146 in VEGF-C induced cell activation. (**a**) Diagrammatic representation of C-terminal truncations of CD146 at the intracellular domain. (**b**) Phosphorylation and expression of ERK1/2 and p38, elicited from VEGF-C156S, were analyzed by WB. HEK293 cells were transfected with plasmids encoding truncated versions of CD146 as shown in (**a**). (**c**–**g**) HDLECs transfected with plasmids encoding CD146-WT and CD146-∆599-646 were subjected to signaling activation assay (**c**), spheroid sprouting assay (**d**), proliferation assay (**e**), tube formation assay (**f**) and transwell migration assay (**g**). VEGF-C was applied at the indicated concentration. Data were expressed as means ± SEM from 3 independent experiments (n = 12 in each group of **c**–**g**,). Significant difference was determined by Student’s *t-*test (*P < 0.05; **P < 0.01; ***P < 0.001; ns, no significant difference). Full-length blots are presented in Supplementary Fig. S4.

To explore the functional domain of CD146 in VEGF-C induced signaling, we generated a series of CD146 constructs truncated at the carboxyl terminal (Fig. 4a) and then transfected them into HEK293 cells. In addition to the full length CD146, either ∆632-646aa (CD146 with 47 amino acids of the intracellular region remaining) or ∆611-646aa (CD146 with 26 amino acids of the intracellular region remaining) preserved the ability of mediating VEGF-C signaling, whereas ∆599-646aa (CD146 with 14 amino acids of the intracellular region remaining) lost its ability to activate p38 and ERK pathways upon VEGF-C156S stimulation for 20 min (Figs 4b and S4). Consistently, overexpression of ∆599-646aa in HDLECs failed to activate VEGF-C156S-induced phosphorylation of p38 and ERK, further confirming that the deleted intracellular region (14-26 amino acid) of CD146 is critical for its function to transduce VEGF-C signaling (Fig. 4c). Based on its capacity to competively combine with VEGF-C but not transduce downstream signaling, ∆599-646aa was used as a dominant negative mutation for endogenous wildtype CD146. We next evaluated the blocking ability of ∆599-646aa in LECs activation. Over-expression of ∆599-646aa in HDLECs blocked all tested VEGF-C156S-induced activities, including sprouting, proliferation, tube formation and migration (Fig. 4d–f). These results indicate that the intracellular region, especially the 14–26 amino acids at the carboxyl terminal of CD146 is critical for transduction of VEGF-C signaling and imply a critical role of CD146 in lymphangiogenesis *in vivo*.

### CD146 mediates sprouting of lymphangiogenesis *in vivo*

**Figure 5:**
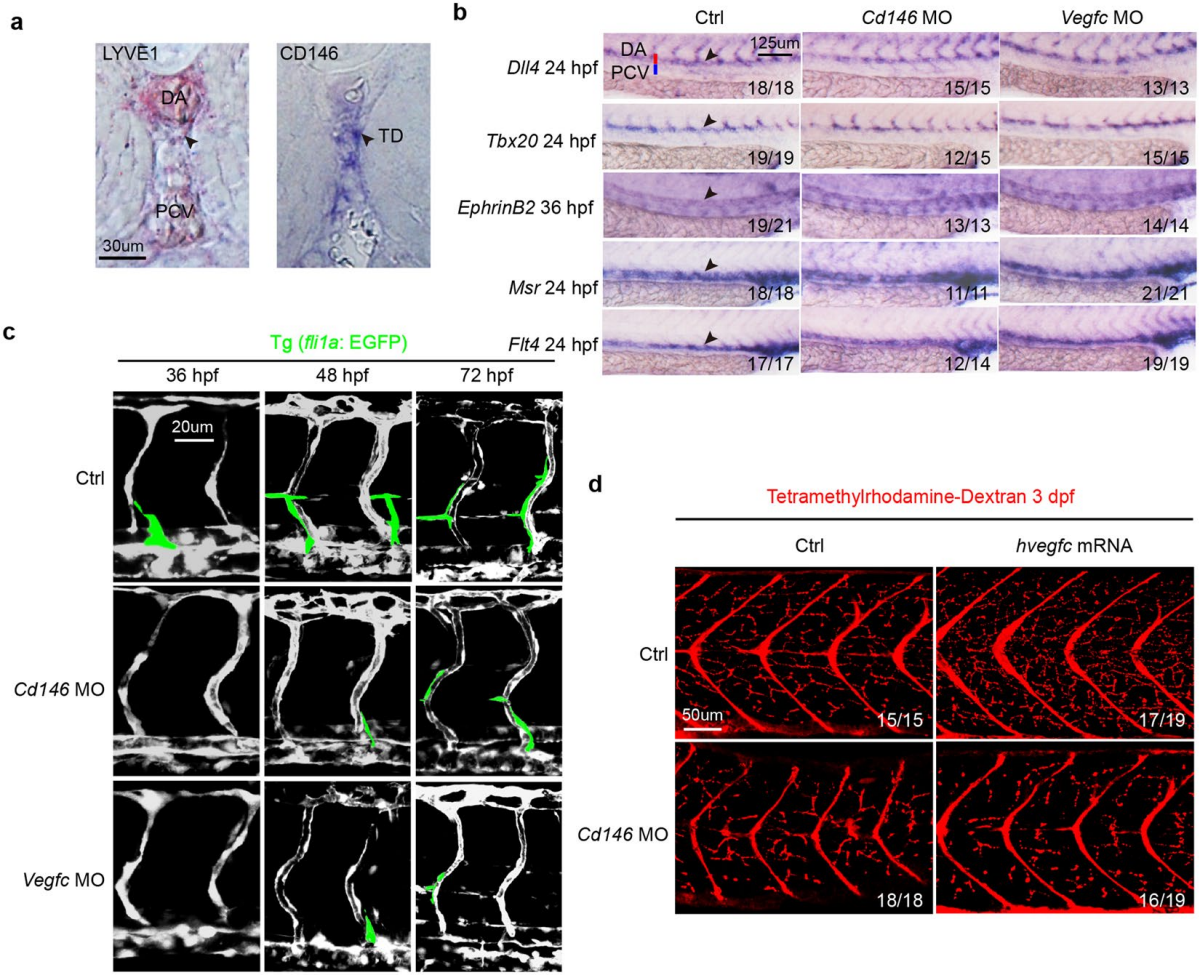
CD146 modulates VEGF-C induced lymphatic development in zebrafish. (**a**) CD146 expression in the zebrafish embryos at 5 dpf was detected by whole-mount *in situ* hybridization (WISH). *lyve1b* was used as positive control. Black arrows indicate the position of TD. DA, dorsal aorta; PCV, posterior cardinal vein; and TD, thoracic duct. Scale bars: 30 μm. (**b**) The effect of knockdown *cd146* or *vegfc* on blood vessels. Bright-field lateral views of the trunk of embryos injected with control, *cd146* or *vegfc* specific MOs. Embryos were stained by WISH for a panel of arterial markers of *dll4*, *tbx20 and ephrinB2* and venous markers of *msr* and *flt4*. Black arrows, blood vessels; DA, dorsal aorta; and PCV, posterior cardinal vein. Scale bars: 125 μm. (**c**) The effect of knockdown *cd146* or *vegfc* on vasculature of blood and lymph. The control, *cd146* or *vegfc* specific MO were injected into the transgenic zebrafish Tg (*fli1a*: EGFP) embryos, respectively. The embryos harvested at 36 hpf, 48 hpf and 72 hpf were analyzed. The confocal images were flanked by redrawing of the vessel contours. Transient lymphangiogenic structures (lymphangiogenic sprouts; PL cells) were labeled with light green. The other vessels (PCV, DA and ISV) were labeled with dark grey. Scale bars: 20 μm. (**d**) The effects of *cd146* knockdown on development of lymphatic capillary. The control or *cd146* specific MO was co-injected with control or human *vegfc* mRNA into the transgenic zebrafish Tg (*fli1a*: EGFP) embryos. Late-phase microangiography was used to visualize lymphatic capillaries in embryos at 3 dpf. Images showed the representative phenotypes of each group and the ratio indicated the embryos with such representative phenotype over the tested embryos. Scale bars: 50 μm.

Next, we used the zebrafish model to explore the *in vivo* function of CD146 in lymphangiogenesis. We have previously reported that *cd146* is expressed in endothelial cells at 24–36 hours post fertilization (hpf) and downregulated at 48 hpf in zebrafish embryos^25^. However, the expression of *cd146* in lymphatic vessels has not been fully characterized. Here we found that *cd146* could be detected in the thoracic duct (TD) at 5 day post fertilization (dpf) by whole mount *in situ* hybridization (WISH) (Fig. 5a), implying that CD146 might be involved in zebrafish lymphangiogenesis and the expression of *cd146* was dynamic in both angiogenesis and lymphangiogenesis. During zebrafish embryogenesis, vascular sprouting occurs at two stages: primary sprouting which gives rise to arterial intersegmental vessels (aISV) and subsequent secondary sprouting including lymphatic sprouting which gives rise to venous intersegmental vessels (vISV) and lymphatic vessels^3^. In lymphatic sprouting, differentiated endothelial cells migrate dorsally to form a transient, non-luminal string of parachordal lymphangioblast (PL) cells and then migrate between the dorsal aorta (DA) and posterior cardinal vein (PCV) to fuse and establish the thoracic duct (TD), the first functional lymphatic vessel.

A *cd146* specific morpholino (MO) was used to knockdown endogenous expression of *cd146* in zebrafish. We found that the PL and TD formation was affected in *cd146* morphants, and the severity degree of PL and TD was dose-dependent of injected *cd146* MO (Fig. S5a,b). As CD146 regulates angiogenesis, we performed a sub-maximal MO dosage assay, which only minimally affected blood vessel development, to avoid angiogenic defects that may secondarily cause lymphatic defects. At a dose of 1.5 ng of *cd146* MO per embryo, arterial-venous differentiation of large axial vessels and primary ISVs was normal, which was similar with that observed in *vegfc* morphants, as evidenced by WISH for arterial markers, including *dll4*, *tbx20* and *ephrinB2*, and venous markers, such as *msr* and *flt4* (Fig. 5b). Interestingly, compared with control embryos of Tg (*fli1a*: EGFP) line with endothelial cells sprouting from PCV at 36 hpf, knockdown of *cd146* or *vegfc* inhibited sprouting at 36 hpf. We also observed that *cd146* and *vegfc* morphants exhibited blockage of secondary (lymphatic) sprouting at 48 hpf and 72 hpf (Fig. 5c). We also found that knockdown of *cd146* did not affect the morphology of zebrafish embryo at 24 hpf and 72 hpf (Fig. S5c). Therefore, knockdown of *cd146* inhibited lymphangiogenesis without delaying the development. We quantified the number of unilateral secondary sprouts and the fraction of venous ISVs. Results showed that knockdown of *cd146* did not affect the fraction of venous ISVs but reduced the number of secondary sprouts, which was caused by inhibition of lymphatic sprouts (Fig. S5e). Furthermore, knocking down *cd146* in Tg (*fli1a*: EGFP) embryos resulted in reduced lymphatic capillaries (Fig. 5d, left panels). In contrast, embryos injected with *vegfc* mRNA showed increased lymphatic capillaries, and such increase was blocked by injection with *cd146* MO (Fig. 5d, right panels). Therefore, our results demonstrated that both CD146 and VEGF-C are key players to ensure that LEC sprouting, the initiating step of lymphangiogenesis, occurs properly during embryogenesis.

### CD146 facilitates TD and PL development in lymphangiogenesis

**Figure 6:**
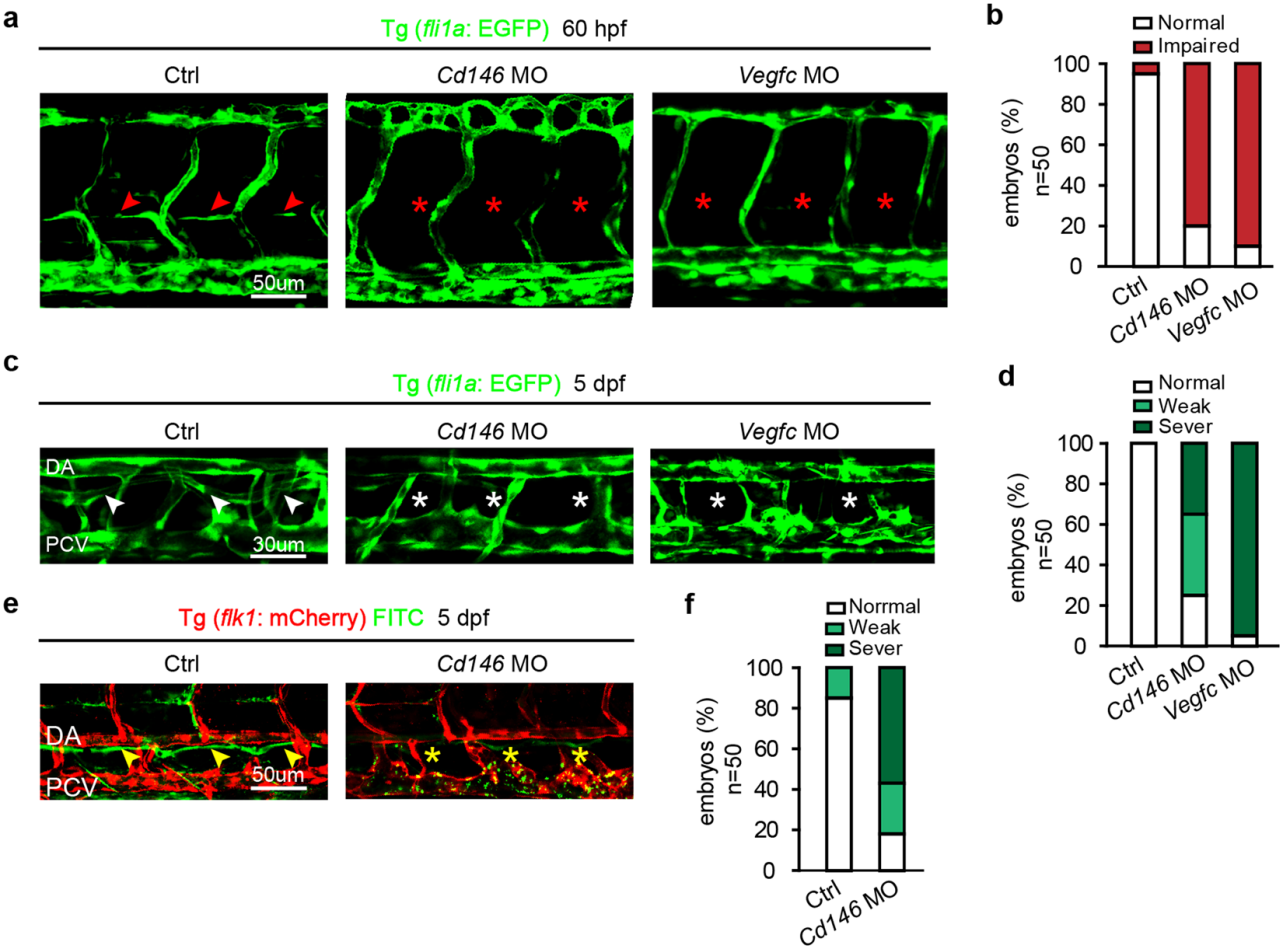
Knockdown of CD146 disrupts lymphangiogenesis in zebrafish. (**a**,**b**) The effects of *cd146* or *vegfc* knockdown on PL formation. Tg (*fli1a*: EGFP) embryos, injected with control, *cd146* or *vegfc* specific MO, were harvested at 60 hpf. Confocal images showed normal PL (red arrows) and impaired PL (red stars) in (**a**). Scale bars: 50 μm. The percentages of embryos with normal PL cells (normal) and impaired PL string formation (impaired PLs) were shown in (**b**). (**c**,**d**) The effects of *cd146* or *vegfc* knockdown on TD formation. Tg (*fli1a*: EGFP) embryos, injected with control, *cd146* or *vegfc* specific MO, were harvested at 5 dpf. Confocal images showed normal TD (white arrows) and abnormal TD (white stars) in (**c**). Scale bars: 30 μm. The percentages of embryos with different phenotypes of TD formation were shown in (**d**). Relative to the normal embryos, the TD length decreased to 30–90% were classified as weak, and those decreased to 10–30% were classified as severe. (**e**,**f**) The effects of *cd146* knockdown on TD function. Tg (*flk1*: mcherry) zebrafish embryos injected with control or *cd146* specific MO and FITC-dextran was injected subcutaneously in the tail of those fishes. The embryos were harvested at 5 dpf. Confocal images showed functional TD (yellow arrows) and dysfunctional TD (yellow stars) in (**e**). Scale bars: 50 μm. The percentages of embryos with different phenotypes of TD formation were shown in (**f**). The criterion used to classify the TD formation was the same as that used in (**d**). In all panels, the head of the embryo faces left and dorsal is up.

To investigate the role of CD146 in the processes of lymphatic development, the formation of PL and TD was examined in *cd146* morphants. PL formation was quantified by measuring its length across 4 somites at 60 hpf in Tg (*fli1a*: EGFP) embryos. In control embryos, 95% of PL strings developed normally (Fig. 6a and b). In contrast, PL impairment was observed in 80% embryos injected with *cd146* MO and in 85% embryos injected with *vegfc* MO (Fig. 6a and b).

TD formation was quantified by measuring its length across 3 somites of 5 dpf embryos. The TD developed as a continuous lymphatic vessel by 5 dpf in control embryos (Fig. 6c and d). In contrast, in *cd146* morphants, 35% of the embryos completely lacked TD by 5 dpf (“severe” defects in Fig. 6c and d) and 40% of the embryos developed into an incomplete string with disconnected segments (“weak” defects in Fig. 6c and d), although 25% of embryos showed normal TD (“normal” in Fig. 6c and d). The defects of TD formation caused by *vegfc* depletion were similar with those resulted from *cd146* deficiency (Fig. 6c and d). Using lymphangiography to detect the draining ability of TD, we found that *cd146* knockdown led to failure of continuous TD vessels (Fig. 6e and f), indicating dysfunctional TD in *cd146* morphants. We also detected the expression of lymphatic markers, including *prox1a*, *prox1b* and *lyve1b* by WISH. Results showed that knockdown of *cd146* reduced the expression of lymphatic markers in TD (Fig. S5f). Taken together, these results demonstrated that CD146 contributes not only to the initial sprouting step but also to the later processes of lymphatic development.

### CD146 regulates lymphangiogenesis independent of vasculogenesis

**Figure 7:**
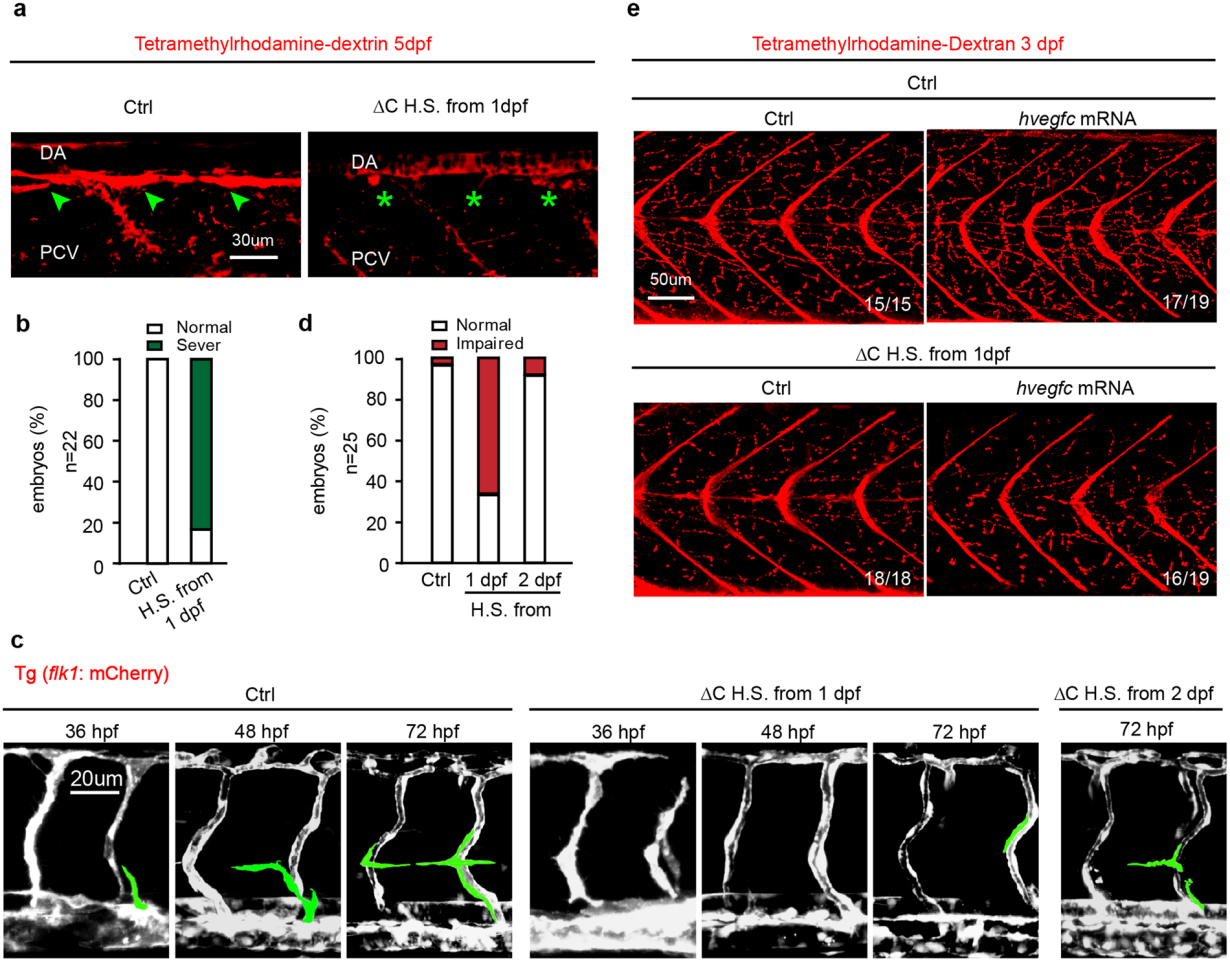
CD146 is essential for lymphangiogenesis independent of angiogenesis. (**a**,**b**) Tg (*fli1a*: EGFP) embryos injected with plasmid of CD146 ∆C-GFP or its empty control, which was induced to express CD146 ∆C-GFP by 37 °C heat-shocking for 30 min. The embryos were harvested at 5 dpf. Tetramethylrhodamine-dextran was used to visualize normal TD (green arrow) and abnormal TD (green stars). The criterion used to classify the TD formation in (**b**) was the same as that used in Fig. 4d. Scale bars: 30 μm. (**c**,**d**) Tg (*flk1*: mcherry) embryos were treated as same as in (**a**). The embryos were harvested at 36 hpf, 48 hpf and 72 hpf, respectively. Vasculature of blood and lymphatic vessels were observed by confocal microscopy. Images were flanked by redrawing of the vessel contours. Transient lymphangiogenic structures (lymphangionenic sprouts; PL cells) were labeled light green and the other vessels (PCV, DA and ISV) were labeled dark grey. Scale bars: 20 μm. (**e**) Tg (*fli1a*: EGFP) embryos were injected with plasmid of CD146 ∆C-GFP or its control plasmid together with control or human *vegfc* mRNA and were heat-shocked to express CD146 ∆C-GFP at 37 °C for 30 min. Late-phase microangiography was used to visualize typical phenotype of lymphatic capillaries in embryos at 3 dpf. The typical ratio was showed in each panel. Scale bars: 50 μm.

To determine the specific role of CD146 in lymphatic sprouting without affecting blood vessel formation, we constructed a plasmid with the cytoplasmic domain of *cd146* deleted in heat shocking system (*hsp70: cd146*∆C-GFP) (Fig. S5d). From 24 hpf (i.e. after arterial-venous differentiation), the Tg (*fli1a*: EGFP) embryos injected with *hsp70: cd146*∆C-GFP were heat-shocked to induce the expression of *hsp70: cd146* ∆C-GFP. Tetramethylrhodamine-dextran was injected subcutaneously to visualize the TD. About 80% of embryos with expression of ∆C-GFP exhibited severe defects of TD formation (Fig. 7a and b), indicating that CD146 regulates the formation of TD specifically, without affecting blood vessels.

To further characterize the role of CD146 in lymphatic sprouting and migration, Tg (*flk1*: mcherry) embryos injected with *hsp70: cd146*∆C-GFP were used to directly visualize these processes. The result showed that lymphatic sprouting from PCV and the final formation of PL were severely blocked in embryos heat-shocked from 24 hpf (before lymphatic sprouting) compared with control embryos, but only slightly affected in embryos heat-shocked from 48 hpf (after lymphatic sprouting) (Fig. 7c and d). More importantly, injection of *vegfc* mRNA into *cd146* morphants cannot rescue *cd146*-dysfuction-caused lymphatic capillary defects (Fig. 7e). Therefore, these results clearly indicate that VEGF-C/CD146 signaling regulates the whole process of lymphangiogenesis, especially lymphatic sprouting, independent of its role in vasculogenesis.

## Discussion

The present study here reveals an unprecedented decoding outcome of VEGF-C signals mediated by CD146 (Fig. S7). We show that, as a novel receptor of VEGF-C, CD146 responds to VEGF-C-elicited signals to mediate sprouting during lymphangiogenesis. Therefore, our results support an indispensable role of CD146 in VEGF-C-induced lymphangiogenesis.

We found the cooperative actions between CD146 and VEGFR3. Knockdown of *cd146* inhibited phosphorylation of p38 and ERK, while knockdown of *vegfr3* inhibited phosphorylation of AKT and ERK. Though CD146 and VEGFR3 activate p38 and AKT, respectively and independently, both of them are required for activation of ERK. Furthermore, the ERK1/2 inhibitor, SCH772984, reduced proliferation and migration of LECs, while the p38 inhibitor, FHPI, mainly reduced sprouting of LECs. Therefore, we reasoned that both CD146 and VEGFR3 regulate proliferation and migration of LECs and both are indispensable. Down-regulation of either molecule abolishes the proliferation and migration. Furthermore, knockdown of both CD146 and VEGFR3 blocks the signaling completely (Fig. 2c), and finally leads to the loss of cell motility (Fig. 1c–f). Together with the direct interaction between CD146 and VEGFR-3 (Fig. 3a–d), these results indicate that the cooperative actions of these two receptors to decode VEGF-C signaling are required for the VEGF-C controlled lymphangiogenesis.

It is known that CD146 is essential in angiogenesis^26, 27^ and VEGFR-3 is important in both angiogenesis and lymphangiogenesis^14, 28^. However, the functions of CD146 in lymphangiogenesis and the exact role of VEGFR-3 in vertebrate lymphatic development remain unknown. Our results provide the first evidence that CD146 and VEGFR-3 play important and distinct roles in VEGF-C induced lymphangiogenesis at different developmental stages. We show that CD146 regulates the whole process of lymphangiogenesis, especially the lymphatic sprouting of LECs from cardinal veins, and that VEGFR-3 mainly regulates proliferation and migration of LECs.

The dual role of CD146 in angiogenesis and lymphangiogenesis raises the question that how CD146 simultaneously regulates these two closely related developmental processes or whether CD146 executes its functions in lymphangiogenesis through its effects on angiogenesis. At 24 hpf of zebrafish embryo, the development of DA and PCV has been largely completed and the lymphatic sprouting from PCV is initiated, while at 48 hpf, the sprouting process is completed and the proliferation and migration processes of lymphatic cells begin. We selected these two time points to induce the expression of cytoplasmic domain-deleted CD146 in heat shocking system (*hsp 70: cd146* ∆C-GFP). The significantly decreased number of lymphatic sprouting cells with heat shock at 24 hpf strongly support that CD146 indeed regulates lymphatic sprouting. The unaffected PL formation with heat shock at 48 hpf further confirms that CD146 regulates the formation of lymphatic vessels, through directly functioning on the lymphatic sprouting process, but not on the proliferation or migration processes.

To identify the binding sites of CD146 with VEGF-C, we also used the blocking antibody of AA98^16, 29^, in the functional assays (Fig. S6). The unchanged phosphorylation of AKT, ERK and p38, and the reduced activity of LECs, indicate that the binding site exists between D4 and D5 domains of the extracellular domain of CD146. Although we demonstrated that the intracellular domains of CD146 is critical for its signaling transduction, the exact amino acid residues responsible for transmitting VEGF-C signals still need to be defined.

CD146 knockdown resulted in decreased number and functional loss of lymphatic vessels, suggesting that CD146 may also play an essential role in lymphatic related diseases. Lymphatic metastasis is a challenge for clinical treatment of tumors and is the leading cause of death from tumors^30, 31^, such as breast cancer^32^, lung cancer^33^, and melanoma^34^. Soluble VEGFR-3^35, 36^, VEGF-C inhibitor^37^, VEGFR-3 antibody^38^, and VEGF-C siRNA^39^ have been used in the treatment of lymphatic metastasis. Based on the fact that VEGF-C is the potent pro-lymphangiogenic factor, it is possible that CD146/VEGF-C is a critical receptor/ligand pair in lymphangiogenesis. For this reason, in the therapeutic regime of pathological lymphangiogenesis, simultaneous targeting VEGF-C, CD146 and VEGFR-3 may have improved efficacy than settings with single targeting.

## Material and Methods

### Antibodies and Reagents

The following antibodies were used in this study: anti-CD146 rabbit polyclonal antibody and anti-CD146 mouse mAb of AA1 were generated in our lab. Anti-β-actin antibody, HRP-conjugated goat anti-mouse and anti-rabbit IgG antibodies were from Sigma-Aldrich. Anti-human IgG Fc and anti-His antibodies were from ZSGB-BIO. Anti-VEGFR-3 antibody and anti-VEGF-C antibody were from Abcam. Anti-AKT, anti-p-AKT, anti-p38, anti-p-p38, anti-ERK and anti-p-ERK antibodies were from Cell Signaling Technology. Goat anti-mouse Alexa Fluor 488 and goat anti-rabbit Alexa Fluor 555 were from Invitrogen.

The following reagents were used: recombinant human VEGFR-3-Fc, human VEGF-C, Fc-CD146 and sCD146 were from Sino Biological. Human VEGF-C156S was from R&D. ERK1/2 inhibitor, SCH772984, and p38 inhibitor, FHPI, were from Selleckchem. Growth factor-reduced Matrigel was from BD Biosciences. Fugene HD, DAPI and protease inhibitor cocktails were from Roche. Protein G sepharose beads was from Santa Cruz. Enhanced Chemiluminescence Assay Kit for WB was from Pierce. Cell Counting Kit-8 (CCK-8) for cell proliferation assay was from Dojindo. Human Fc, Carboxymethylcellulose for spheroid assay and Fluorescein isothiocyanate-dextran average molecular weight 2000 KD and Tetramethylrhodamine-dextran average molecular weight 70 KD were from Sigma-Aldrich.

### Cell line

Mouse brain endothelial BEND3 cells and Human Embryonic Kidney (HEK293) were obtained from the ATCC. Mouse axillary lymph node endothelial SVEC4-10 cell line was a gift from Dr. Mingzhao Zhu. Human umbilical vein endothelial cells (HUVEC) were from CellSystems Biotechnolegie Vertrieb. Primary Human Dermal Lymphatic Endothelial Cells (HDLECs) were obtained from ScienCell. All cells were grown at 37 °C and 5% CO_2_.

### Co-immunoprecipitation

HDLEC or HEK293 cells co-transfected with plasmids encoding the full-length CD146, VEGF-C or the truncation mutants using Fugene HD for 48 hours were lysed in ice-cold RIPA buffer (150 mM NaCl, 50 mM Tris, pH 8.0, 0.1% SDS, 0.5% deoxycholate, 0.1% NP-40, 1 mM PMSF, protease inhibitor cocktails). Then the cell lysates were incubated with the control mIgG, anti-CD146 mAb AA1 or anti-VEGF-C mAb overnight at 4 °C. The immunoprecipitation was carried out using protein G sepharose beads. The cell lysates and precipitates were analyzed by WB using chemiluminescence imaging system (ChemiScope 3400; Clinx, China).

### Pull-down assay

For pull-down assay, purified human Fc, Fc-CD146 or Fc-VEGFR-3 (200 ng/ml each) was incubated with recombinant hVEGF-C (200 ng/ml) for 1 h at 4 °C in HEPES buffer (0.05 M HEPES, 0.15 M NaCl, 0.001% Tween 20, pH 7.4) and then immobilized on protein G sepharose beads for 1 h. After three washes in HEPES buffer, the bound proteins were analyzed by WB.

### Cell surface-binding assay

Plasmid encoding the full-length CD146 was transfected into HEK293 cells for 48 h and incubated with cell culture (VEGF-C conditional medium) for 1 h. After fixation with 4% paraformaldehyde at room temperature for 15 min, cells were incubated with anti-VEGF-C antibody and anti-CD146 antibody overnight at 4 °C, before detection with goat anti-mouse Alexa Fluor 488 and goat anti-rabbit Alexa Fluor 555. Images of spheroids were captured with a confocal microscope (Olympus FLUOVIEW FV 1000 with an Olympus IX81 digital camera).

### RNA interference

CD146- and VEGFR-3-specific siRNAs were synthesized by Invitrogen using sequences as previously described^26^. HDLECs were transfected using Fugene HD and functional or signaling assays were carried out 48 h post transfection.

### LEC spheroid sprouting assay

For the LEC spheroid assay^40^, HDLECs were allowed to aggregate in round-bottom 96-well plates precoated with 0.2% (wt/vol) carboxymethylcellulose for 24 hours (3000 cells per spheroid). The spheroids were subsequently embedded in 20% matrigel-containing and cultured in the presence or absence of VEGF-C156S (200 ng/ml) for 8 h. Images of spheroids were captured under a microscope (Eclipse model TS100; Nikon). Images were captured with a CCD color camera (model KP-D20AU; Hitachi).

### LEC proliferation assay

Cell proliferation assay was carried out using a CCK-8 kit. Briefly, HDLECs were suspended and seeded into a 96-well plate. Human VEGF-C156S directly added to the culture medium. After culturing for 48 h, cells were incubated with 100 μl of 10% CCK-8 reagent for 1 h at 37 °C. The color reaction was measured at 450 nm with a BioRad ELISA reader (Richmond, CA, USA).

### LEC tube formation assay

In tube formation assay, 96-well culture plates were coated with Matrigel to a total volume of 60 μl per well and allowed to solidify for 30 min at 37 °C. VEGF-C156S was added directly to the HDLEC suspensions in complete DMEM medium, and the cells were then seeded into the corresponding wells to a total number of 1 × 10^4^ cells per well in 96-well plate. Cells were incubated at 37 °C for 10 h. Tube formation was observed under an inverted microscope (Eclipse Model TS100; Nikon, Japan) and tube length was measured using NIH Image J software.

### LEC migration assay

HDLECs migration was examined using a modified Boyden chamber assay (8 μm pore size; Costar, Corning, USA). HDLECs were suspended in fresh serum-free medium and seeded in upper chambers of the transwell plate to a total number of 1 × 10^4^ cells per chamber. Lower chambers contained fresh medium containing 10% fetal calf serum (FCS). After 12 h incubation at 37 °C, cells remaining at the upper surface of the membrane were removed using a swab, whereas the cells migrated to the lower membrane surface were fixed with 4% PFA and stained with 0.1% crystal violet solution. The number of cells migrating through the filter was scored.

### Zebrafish

Adult zebrafish including Tubingen, Tg (*fli1a*: EGFP)^41^ and Tg (*flk1*: mcherry)^42^ were used in this study. Zebrafish embryos were obtained by natural spawning of adult fish. Embryos were raised and maintained at 28.5 °C and staged morphologically as described previously. For This study was approved by the Ethical Review Committee of Institute of Zoology, Chinese Academy of Sciences, China. All methods in this work were performed in accordance with the People’s Republic of China regulation of experimental animals.

### Morpholinos, mRNA synthesis and microinjection in zebrafish

The *cd146*-MO (5′-AGCAGTGCGGTGTAGGTCATTTCTC-3′) and *vegfc*-MO (5′-GAAAATCCAAATAAGTGCATTTTAG-3′) were used as previously reported^25^. For mRNA synthesis, human *vegfc* full-length coding sequence cDNA (CDS) was cloned into the pCS2 + vector. Capped mRNA was synthesized using the mMACHINE SP6 Kit (Ambion). For overexpression of cytoplasmic domain-deleted CD146 under the *hsp-70* promoter, the full-length CDS of zebrafish cytoplasmic domain-deleted *cd146* was cloned into pDONR221 vector by BP reaction then was subcloned into a vector with *hsp-70* promoter and GFP reporter by LR reaction (MultiSite Gateway Technology, Invitrogen). MOs, mRNAs and *hsp70-cd146*∆C-GFP plasmid were injected into one-cell stage zebrafish embryos at the yolk/blastomere boundary.

### Zebrafish confocal imaging

Embryos for microscopic observation and photography were prepared as previously^43^ described. Fluorescent images were taken using Nikon A1 confocal microscopy.

### Whole mount *in situ* hybridization (WISH)

WISH of zebrafish embryos was performed as described previously^44^ using probes of *cd146*, *lyve1b*, *dll4*, *tbx20*, *ephrinB2*, *msr* and *flt4*. The embryos were observed with a Nikon C-DSS230 stereo-microscope, and the images were taken with a Nikon DS-U2 camera using NIS-Elements (version F3.0).

### Microangiography and lymphangiography

Microangiography was performed by subcutaneously injection of Low-molecular-weight rhodamine-dextran to visualize 3-dpf Tg (*fli1a*:EGFP) embryos^45^. Lymphangiography was performed by subcutaneously injection of high-molecular-weight -FITC-dextran to visualize 5 dpf Tg (*flk1*: mcherry) embryos^46^.

### Statistical analysis

Unless specified otherwise, all data represent at least 3 independent experiments in each assay. Results are expressed as the mean ± SEM. One-way ANOVA with Turkey post hoc tests were used to compare differences between groups in various experiments.

All data generated during this study are included in this published article.

## Electronic supplementary material

Supplementary PDF File
