# Supplementary material for: CD146 is required for VEGF-C-induced lymphatic sprouting during lymphangiogenesis
Source: Sci Rep. 2017 Aug 7;7:7442. doi: 10.1038/s41598-017-06637-7 (PMC5547131; doi:10.1038/s41598-017-06637-7)
Supplement: Supplementary file 1 — Supplementary PDF File [file 41598_2017_6637_MOESM1_ESM.pdf]

CD146 is required for VEGF-C-induced lymphatic sprouting during lymphangiogenesis

Huiwen Yan[1,3,#], Chunxia Zhang[2,3,#], Zhaoqing Wang[1], Tao Tu[1], Hongxia Duan[1], Yongting Luo[1], Jing Feng[1], Feng Liu[2]*, Xiyun Yan[1, 3]*

[1]Key Laboratory of Protein and Peptide Pharmaceuticals, Institute of Biophysics, Chinese Academy of Sciences, Beijing 100101, China

[2]State Key Laboratory of Membrane Biology, Institute of Zoology, Chinese Academy of Sciences, Beijing 100101, China

[3]College of Life Sciences, University of Chinese Academy of Sciences, Beijing 100049, China

[#]These authors contributed equally to this work

*Correspondence: Xiyun Yan[a], Feng Liu[b]

[a]E-mail: yanxy@ibp.ac.cn

[b]E-mail: liuf@ioz.ac.cn

Supplementary information

Supplementary Figure S1

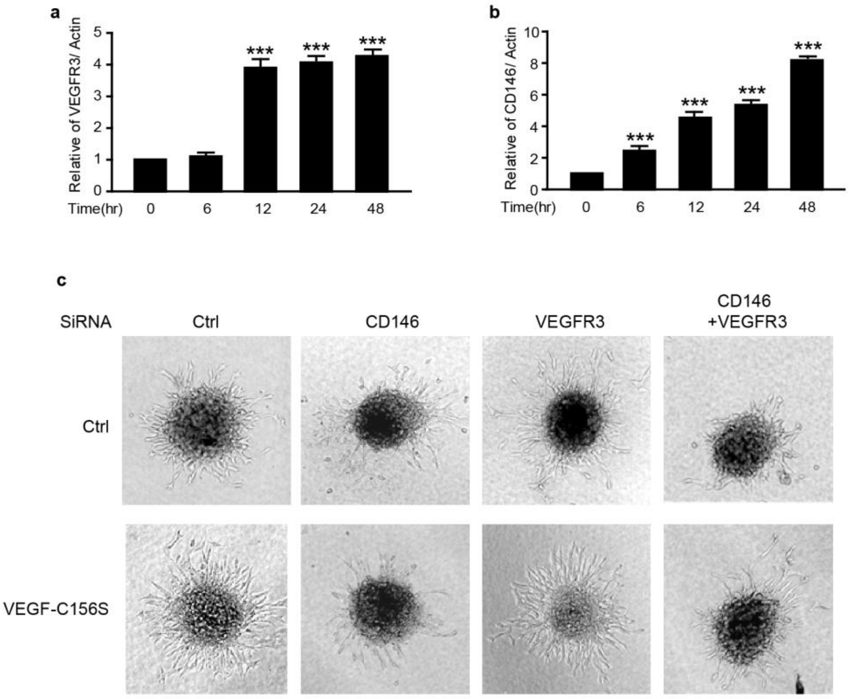

Supplementary Figure S1. Quantification of the expression of VEGFR-3 and CD146 in HDLECs induced by VEGF-C156S for different time intervals. (**a**) The VEGFR-3 quantification was determined by measuring the band (Fig. 1b) density and then normalizing against internal controls. The signal from control cells was put to one. Results were presented as the means ± SEM of normalized values from 3 independent assays. (**b**) The CD146 quantification was determined. ***, *P*< 0.001; compared with control group. (**c**) The representative images of original cell culture to showed the effect of CD146 KD on LEC sprouting.

Supplementary Figure S2

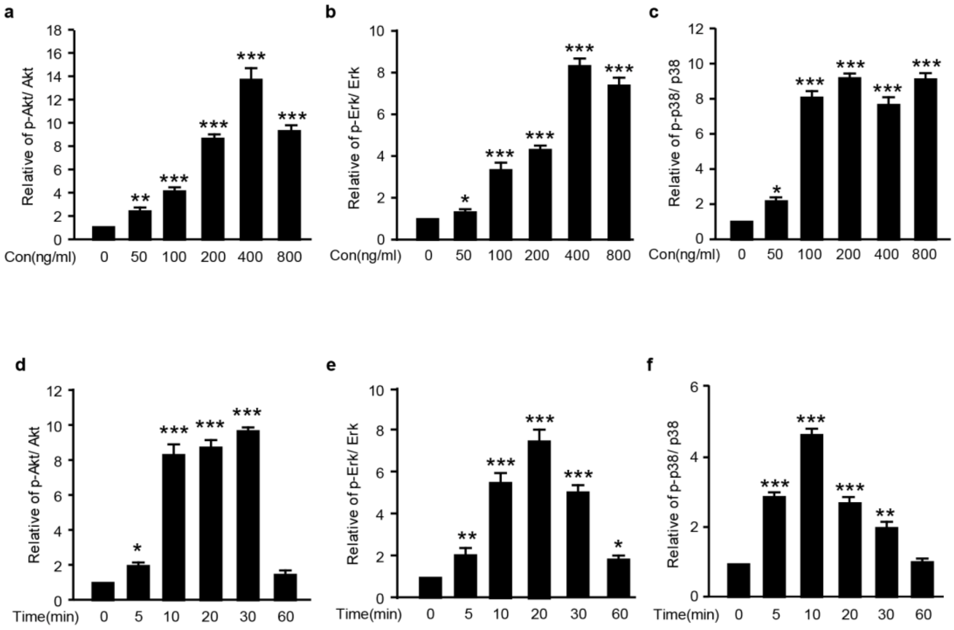

Supplementary Figure S2. Quantification of the phosphorylation of AKT, ERK and p38 induced by VEGF-C-ΔNΔC156S. (**a-c**) The phosphorylation of AKT, ERK and p38 were determined in HDLECs induced by VEGF-C-ΔNΔC156S for different dose intervals. The band (Fig. 2a) density (mean ± SEM) was measured from at least 3 independent immunoblots and was normalized to the paired total. The signal from control group was put to one.*, *P*<0.05, **, *P* <0.01, ***, *P*< 0.001, compared with control group.

Supplementary Figure S3

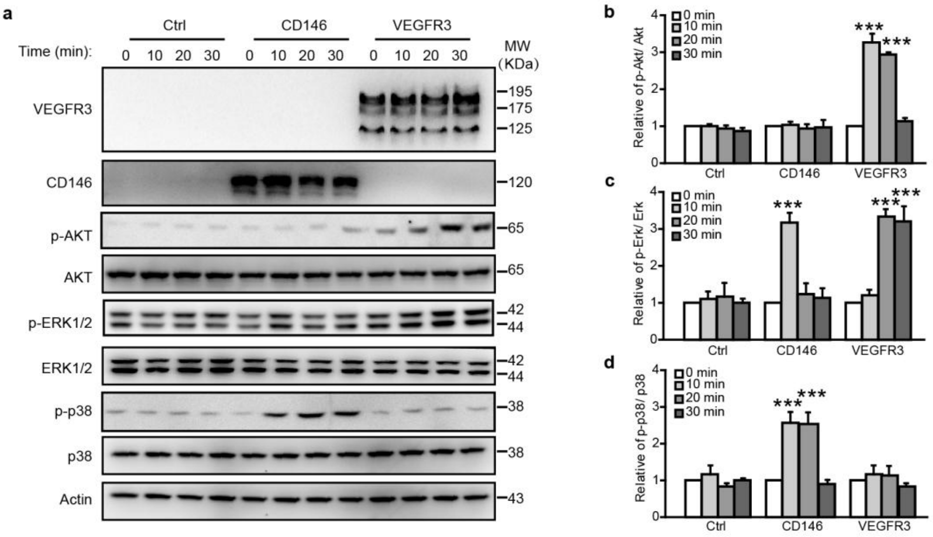

Supplementary Figure S3. Overexpression effects of CD146 or VEGFR-3 on VEGF-C-induced signaling activation. (**a**) HEK293 cells, transfected with empty plasmid, CD146 expression plasmid or VEGFR-3 expression plasmid, were stimulated by VEGF-C. (**b-d**) The phosphorylation of AKT, ERK and p38 were determined in HEK293, overexpressing CD146 or VEGFR-3 respectively. The band density was corresponded to (a). *** $P < 0.001$, compared with control group.

Supplementary Figure S4

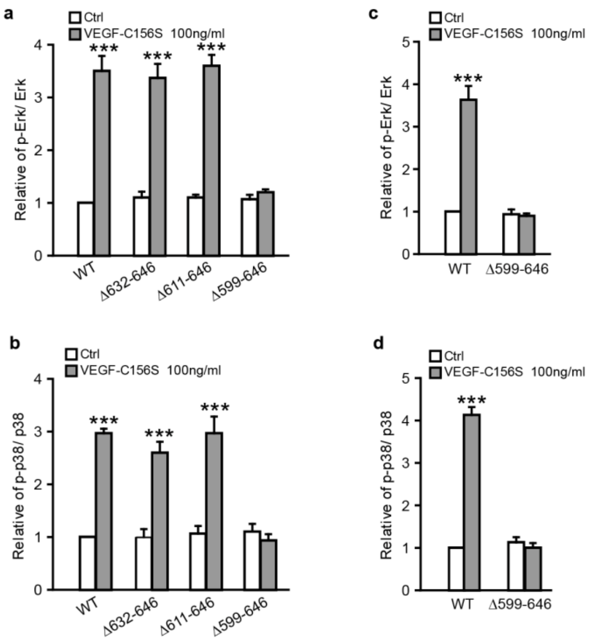

Supplementary Figure S4. Quantification of the phosphorylation of ERK and p38 in cells with overexpresion of series truncated versions of CD146. (**a, b**) The phosphorylation of ERK and p38 were determined in HEK293 transfected with plasmids encoding truncted versions of CD146. The band density was corresponded to Fig. 4b. (**c, d**) The phosphorylation of ERK and p38 were determined in HDLEC transfected with plasmids encoding CD146-WT or CD146-Δ599-646. The density was corresponded to the WB bands in Fig. 4c. ***, P<0.001, compared with control group.

Supplementary Figure S5

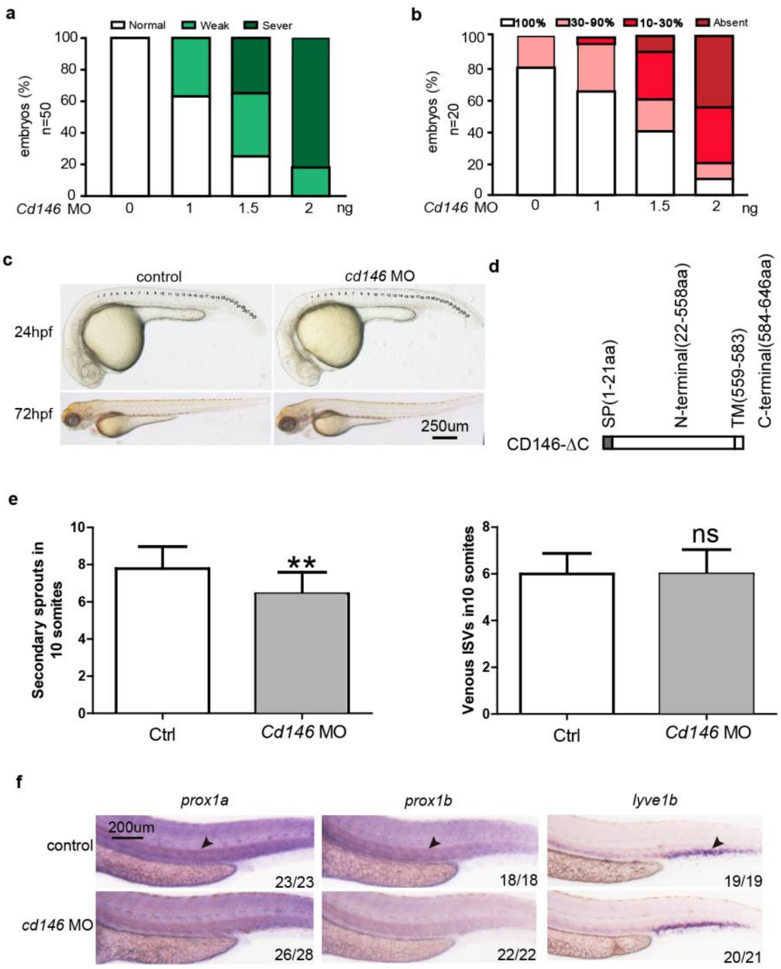

Supplementary Figure S5. Knockdown of CD146 disrupts PL and TD formation in zebrafish. (**a**) Quantification of the TD formation defects after injection of different dose of *cd146* MO at 5 dpf. Relative to the normal embryos, the TD length decreased to 30%-90% were classified as weak, and those decreased to 10%-30% were classified as sever. (**b**) Quantification of the PL formation defects after injection of different dose of *cd146* MO at 60 hpf. Percentages of embryos displaying complete lack of PL, PL formation over 10%-30% or 30%-90% of its normal length, and a normal PL were represented for each treatment group. (c) The effect of knockdown *cd146* on morphology of zebrafish embryo at 24hpf and 72hpf. Bright-field side views of the whole embryos injected with control or *cd146* specific MO. Scale bars: 250 μm. (d) Diagrammatic representation of C-terminal domain deleted CD146.

(e) Quantificaiton of the number of unilateral secondary sprouts (left) and the fraction of venous ISVs, identified by their connection to the PCV upon confocal screening. **, $P<0.05$, compared with control group. (f) The effect of knockdown *cd146* on lymphatic vessels. Bright-field side views of the trunk of embryos injected with control or CD146 specific MO. Embryos were stained by WISH for a panel of lymphatic markers of *prox1a, prox1b and lyve1b*. Black arrows, TD. Scale bars: 200 μm.

Supplementary Figure S6

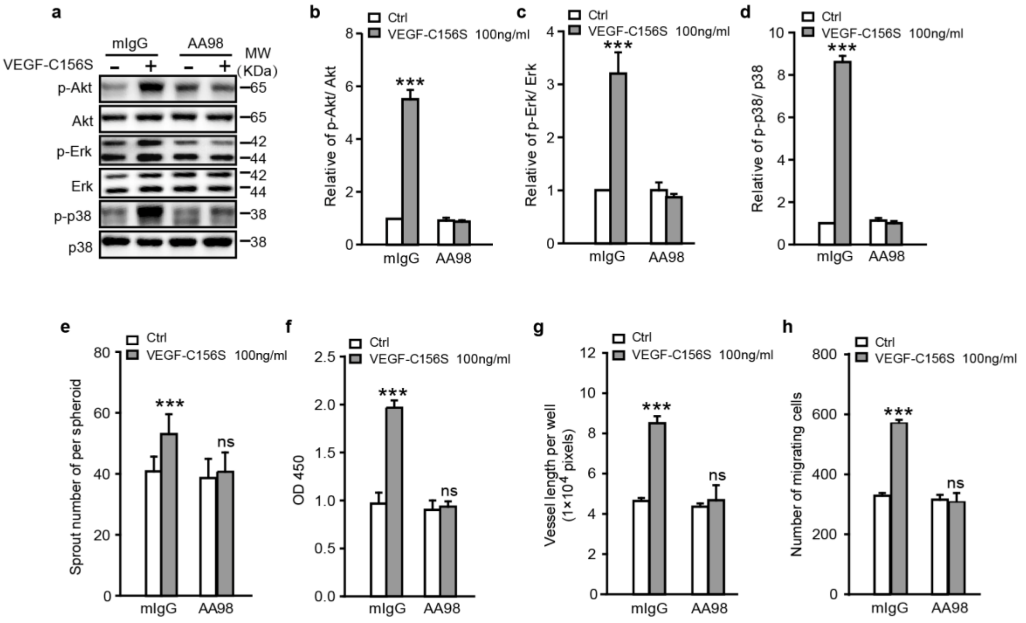

Supplementary Figure S6. The CD146-specific antibody AA98 blocks VEGF-C induced HDLEC activation. HDLECs were used in signaling activation assays (**a**), spheroid sprouting assay (**e**), proliferation assay (**f**), tube formation assay (**g**), transwell cell migration assay (h) in the presence of anti-CD146 mAb AA98 or the control mIgG (100 μg/ml). VEGF-C was used at the concentrations as indicated. Quantification of the phosphorylation of AKT, ERK and p38 were showed in (**b-d)**. For (**b-h**), n=3 in each group. Data represent 3 independent experiments (means ± SEM). ***, *P*<0.001, “ns” indicates no significant changes observed (one-way ANOVA with Turkey post-hoc tests).

Supplementary Figure S7

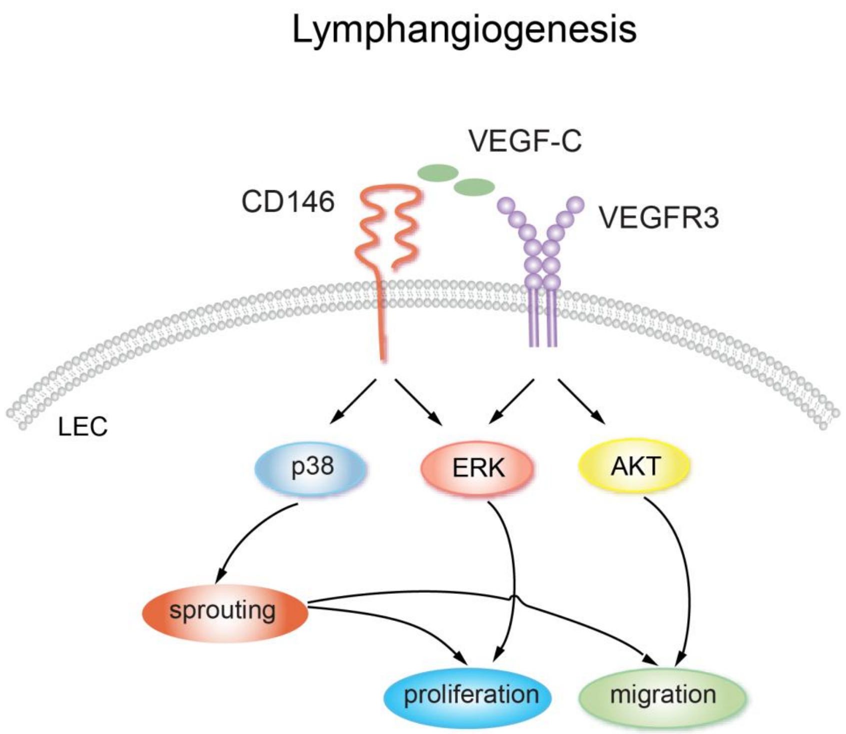

Supplementary Figure S7. Schematic of CD146 involved signal pathways in lymphangiogenesis.

Supplementary Figure S8

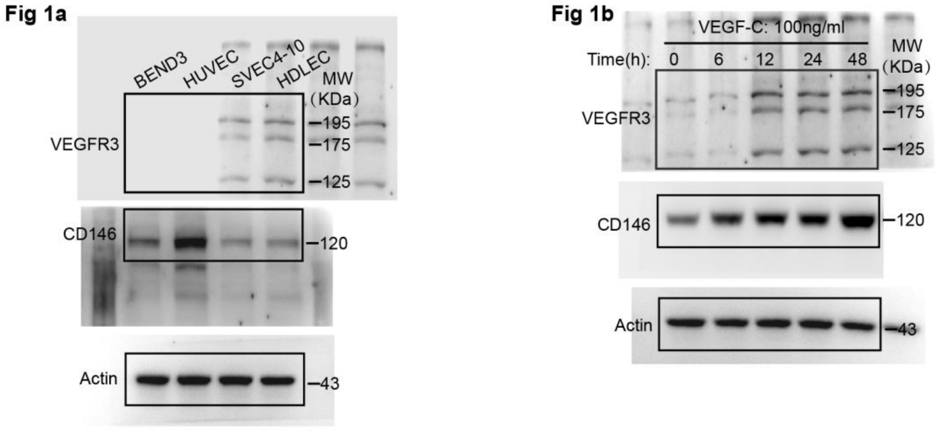

Supplementary Figure S8. Full-length western blot images of Figures 1-4.

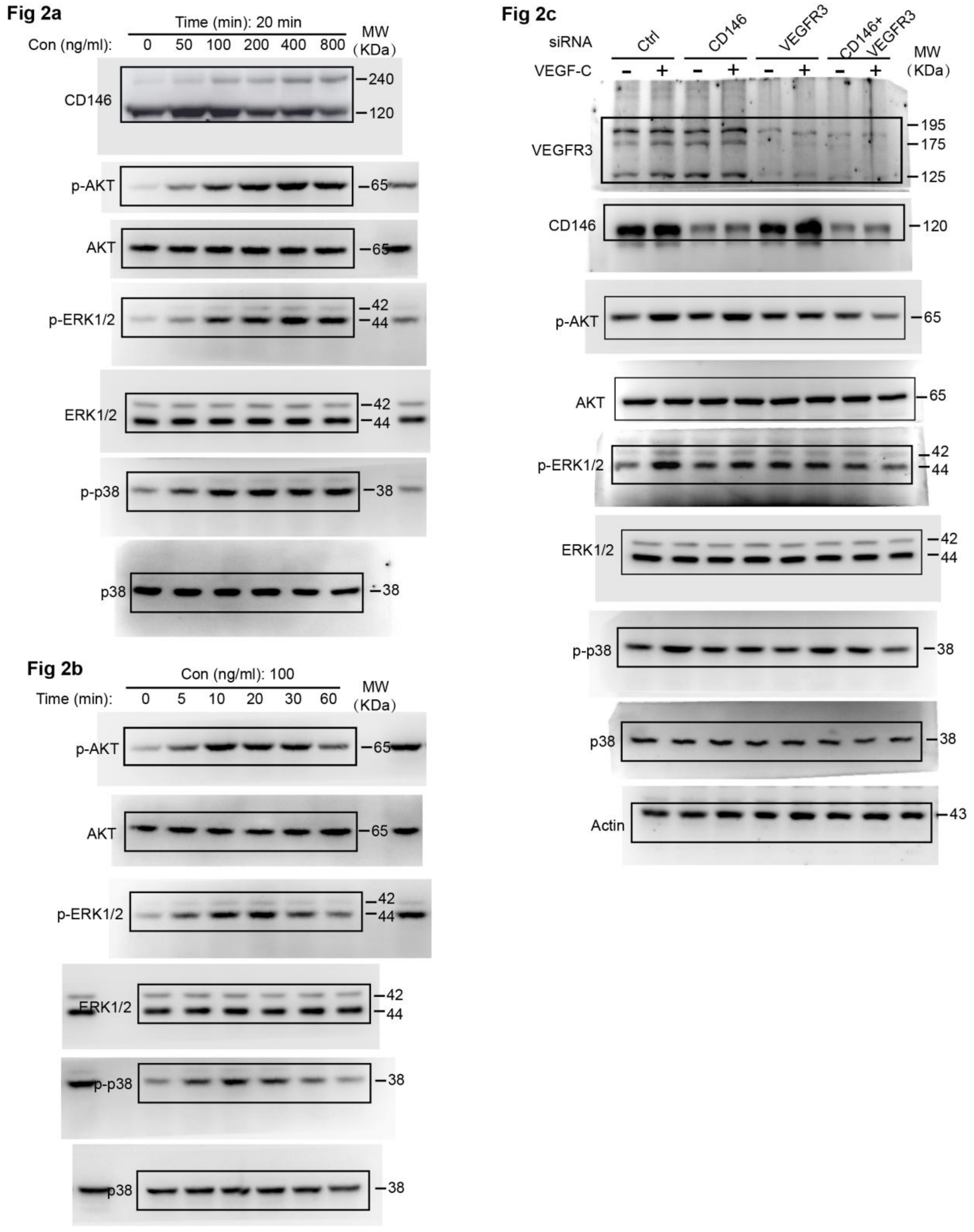

Supplementary Figure S8 continued

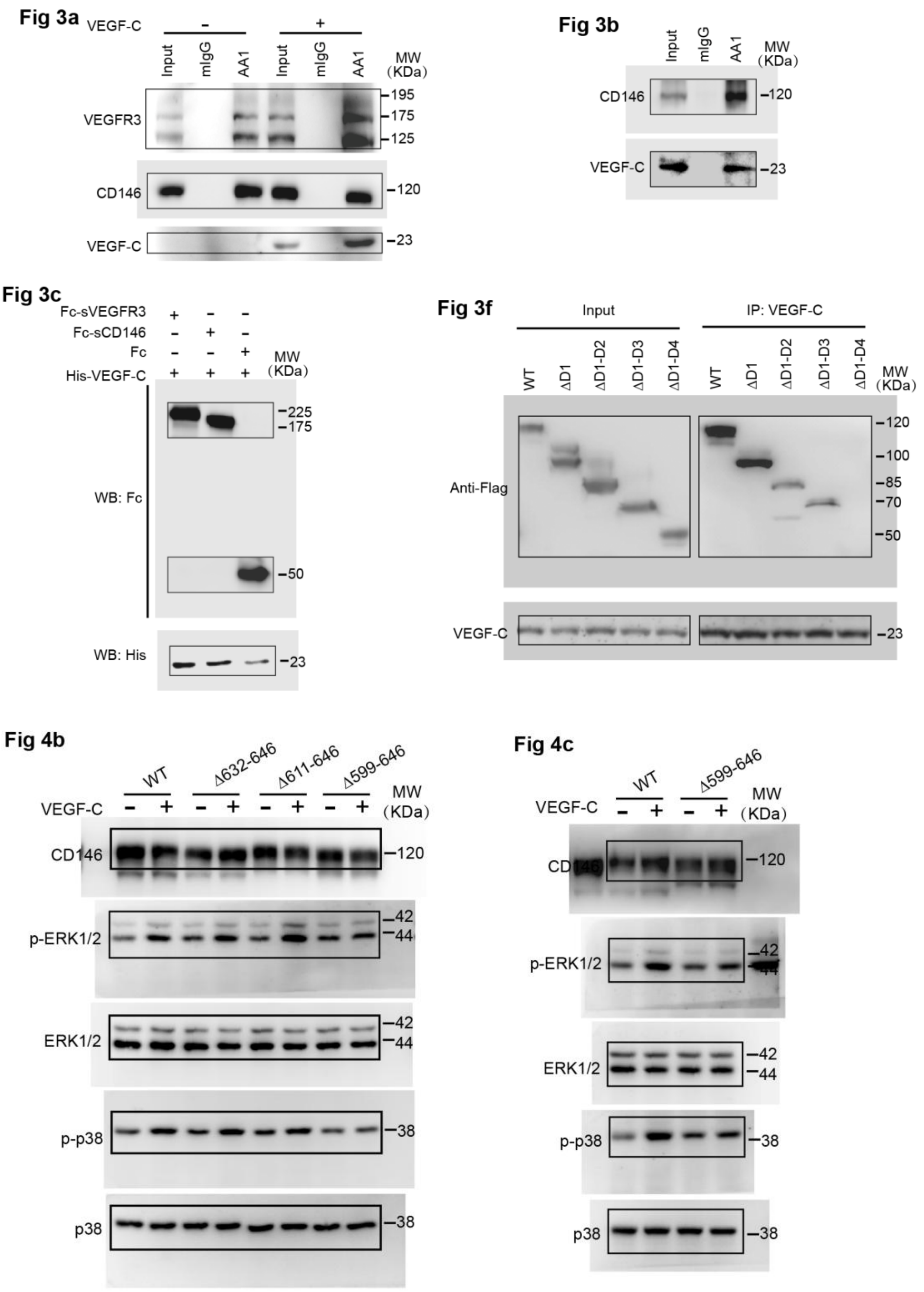

Supplementary Figure S8 continued
